# How Much Remains Undetected? Probability of Molecular Detection of Human *Plasmodia* in the Field

**DOI:** 10.1371/journal.pone.0019010

**Published:** 2011-04-28

**Authors:** Cristian Koepfli, Sonja Schoepflin, Michael Bretscher, Enmoore Lin, Benson Kiniboro, Peter A. Zimmerman, Peter Siba, Thomas A. Smith, Ivo Mueller, Ingrid Felger

**Affiliations:** 1 Swiss Tropical and Public Health Institute, Basel, Switzerland; 2 University of Basel, Basel, Switzerland; 3 Papua New Guinea Institute of Medical Research, Goroka, Eastern Highlands Province, Papua New Guinea; 4 Center for Global Health and Diseases, Case Western Reserve University, Cleveland, Ohio, United States of America; Kenya Medical Research Institute - Wellcome Trust Research Programme, Kenya

## Abstract

**Background:**

In malaria endemic areas, most people are simultaneously infected with different parasite clones. Detection of individual clones is hampered when their densities fluctuate around the detection limit and, in case of *P. falciparum*, by sequestration during part of their life cycle. This has important implications for measures of levels of infection or for the outcome of clinical trials. This study aimed at measuring the detectability of individual *P. falciparum* and *P. vivax* parasite clones in consecutive samples of the same patient and at investigating the impact of sampling strategies on basic epidemiological measures such as multiplicity of infection (MOI).

**Methods:**

Samples were obtained in a repeated cross-sectional field survey in 1 to 4.5 years old children from Papua New Guinea, who were followed up in 2-monthly intervals over 16 months. At each follow-up visit, two consecutive blood samples were collected from each child at intervals of 24 hours. Samples were genotyped for the polymorphic markers *msp2* for *P. falciparum* and *msp1*F3 and MS16 for *P. vivax*. Observed prevalence and mean MOI estimated from single samples per host were compared to combined data from sampling twice within 24 h.

**Findings and Conclusion:**

Estimated detectability was high in our data set (0.79 [95% CI 0.76–0.82] for *P. falciparum* and, depending on the marker, 0.61 [0.58–0.63] or 0.73 [0.71–0.75] for *P. vivax*). When genotyping data from sequential samples, collected 24 hours apart, were combined, the increase in measured prevalence was moderate, 6 to 9% of all infections were missed on a single day. The effect on observed MOI was more pronounced, 18 to 31% of all individual clones were not detected in a single bleed. Repeated sampling revealed little difference between detectability of *P. falciparum* and *P. vivax*.

## Introduction

Detection of malaria parasites is essential for many malariological investigations. For instance, the detailed maps of global malaria risk created by the Malaria Atlas Project (MAP) use parasite prevalence as the main indicator for transmission [1], [2], [3], [4]. Prevalence is also the key malariological measure assessed in Malaria Indicator Surveys (MIS) [5], [6]. Within populations, the identification of individual parasite clones by PCR based genotyping techniques has substantially increased knowledge of the infection dynamics of malaria by providing estimates of multiplicity of infection, incidence and clearance rates [7], [8], [9], [10]. In addition, it allows classification of drug failures into recrudescences and new infections.

However, all DNA based techniques for detection of malaria parasites in a blood sample are imperfect. Parasites can remain undetected because their densities fall below the detection limit of the diagnostic technique. PCR based methods generally have a better detection limit compared to microscopy [11], but both methods likely miss a proportion of clones.

Several biological factors add to the probability of low numbers of parasite in the blood stream and subsequently of missing clones. In *P. Falciparum* infections late blood stage forms cytoadhere to the endothelial wall of blood vessels. This sequestration of *P. Falciparum* in deep organs lasts for 24–28 hrs of the 48 hrs blood stage cycle. During this period parasites are absent from the peripheral circulation and escape detection. A parasite clone defined by a shared genotype and by common ancestry is completely absent only if all individual parasites belonging to this brood are tightly synchronized. By rare chance a clone might be superinfected by another clone sharing the same genotype. The probability of this occurring is determined by the resolution of the typing scheme and the mean multiplicity of infection in the study area [12].

In blood stage schizogony a single schizont divides into numerous merozoites [13] within a few hours. This has particular implications for *P. Vivax*, because these parasites usually divide synchronously. As a result the number of DNA copies of a given clone may drastically increase from one day to another. In addition, parasite densities of *P. Vivax* are usually lower than those of *P. Falciparum* [14], [15]. Taken these factors together, imperfect detection of *P. Vivax* clones is likely, however, only for *P. Falciparum* this has been suspected because of sequestration.

Besides these issues of parasite biology, also methodological constraints may add to the risk of missing clones. These causes equally apply for both parasite species. In multi-clonal infections minority variants constituting a small proportion of the total parasite load, might be missed by PCR-based detection, because in competition for primers or other constituents of the reaction mix, they are outcompeted by the more abundant clones.”

Although sequestration of *P. Falciparum* has been reported several decades ago [16], [17], low parasite densities in *P. Vivax* and the sensitivity threshold of both light microscopy and PCR-based diagnostic methods have been well studied [18], [19], [20], the consequences of imperfect detectability for estimates of prevalence and other epidemiological parameters have often not been addressed adequately.

The daily dynamics of *P. Falciparum* clones has been investigated previously in a longitudinal study with daily follow up bleeds collected over a period of 14 days from 20 children from Tanzania [21]. A complex dynamics of *P. Falciparum* clones was observed. The composition of infecting clones found in a single host was found to be unstable over time or even changing from one day to another [21]. In a drug efficacy trial in Tanzania children were sampled on two consecutive days and the results were compared to the standard protocol with single bleeds. An increased number of multiple clone infections and additional recrudescence cases were detected [22]. Several studies have described statistical models to estimate infection dynamics of *P. Falciparum* allowing for this imperfect detection [23], [24], [25], [26]. The model by Sama et al. [23] was applied for a longitudinal field study in Ghana, where blood samples were collected once at each of 6 cross sectional surveys conducted in 2-monthly intervals. Statistical models indicated that at any time of sampling on average only 47% of all parasite clones present in a host were detected by genotyping [23].

Back in 1966 Garnham reported a slight tendency of late *P. Vivax* stages to retreat from the peripheral circulation [27], but sequestration is generally thought to be absent in *P. Vivax*. This view was questioned only recently when cytoadherence of *P. Vivax* was shown in vitro [28]. Few studies have addressed the infection dynamics of individual *P. Vivax* clones [7]. Daily fluctuations in detectability of *P. Vivax* clones have not yet been investigated.

To obtain a more precise picture of the parasite population present in a host, blood sampling should be repeated within short time intervals. However, collection of consecutive blood samples from a study participant will translate into considerably increased efforts in the field and laboratory, added costs, and additional discomfort for participants. Limited knowledge has been gathered so far on the effect of such a sampling scheme on epidemiological measures, such as prevalence or multiplicity of infection.

**Figure 1:**
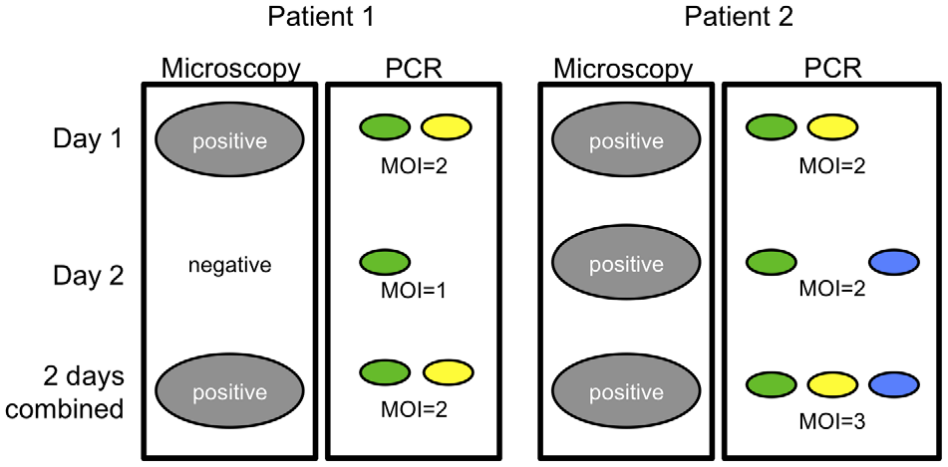
Dynamics of parasite clones over 24 hours. Schematic overview of possible outcomes of 24 h bleeds. A sample is positive as soon as a parasite is detected on either day. Different colors of PCR results indicate different clones detected. The combined multiplicity of infection includes all clones detected in two corresponding bleeds.

Here we present data from a large set of paired samples that were collected 24 hours apart. Within this time interval re-infection with a new parasite clone is very unlikely and may be ignored. Samples were derived from a cohort study performed in Papua New Guinean children 1 to 4.5 years of age living in an area highly endemic for both *P. Falciparum* and *P. Vivax*. Parasites of both species were genotyped to calculate the detectability of infection and to investigate the benefit of collecting 24 h bleeds on basic and molecular measures of epidemiology such as prevalence and multiplicity of infection. Figure 1 gives a schematic overview on the effect of combining results obtained on two consecutive days.

## Methods

### Ethics Statement

Scientific approval and ethical clearance for the study was obtained from the Medical Research and Advisory Committee (MRAC) of the Ministry of Health in PNG and from the Ethikkommission beider Basel in Switzerland. Informed written consent was sought from all parents or guardians prior to recruitment of each child.

### Field survey and patients

This study was conducted in a rural area near Maprik, East Sepik Province, Papua New Guinea. A detailed description of the study was given previously [15]. Briefly, 269 study participants were enrolled at an age of 1 to 3 years starting in March 2006 and regular follow-up visits were conducted over a period of 16 months until July 2007. At seven time points separated by 8-weekly intervals two consecutive 250 µl finger prick blood samples were collected at intervals of 24 hours (in the following termed: 24 h bleed) from each study participant. Two blood slides were made and a rapid diagnostic test was performed upon presentation of malaria symptoms. Antimalarial treatment with Coartem® (Novartis, Switzerland) was administered upon a positive test plus iron and folate supplementation if haemoglobin levels were <7.5 g/dl. Children that were treated on day 1 of the 24 h bleed were excluded from the analysis (n = 97). Over the 16 months follow up period, study participants adhered to 93 to 94% of all study visits.

### Laboratory procedures

Diagnosis of the different malaria species was performed in parallel by two methods, light microscopy (LM) and post-PCR Ligase Detection Reaction (LDR) [29]. Only samples positive by light microscopy and/or LDR were genotyped for our markers.

All finger prick blood samples were separated into plasma and cells. DNA was extracted from cell pellets using QIAamp® 96 DNA Blood Kit (Qiagen, Australia) according to the manufacturer's instructions. All samples were genotyped for the polymorphic marker gene merozoite surface protein 2 (*msp2*) by use of capillary electrophoresis (PCR-CE) for fragment sizing as previously described by Falk et al. [18] with some minor changes and adaptations of PCR conditions for highly purified DNA as described by Schoepflin et al. [12].

*P. vivax* genotyping was performed as described previously [30] with the following modifications: A multiplex primary PCR was done with the primers for the 2 markers *msp1*F3 and MS16 followed by individual nested PCRs for *msp1*F3 and MS16. Primary PCR was performed in a volume of 20 µl containing 1 µl template DNA, 0.25 µM of each primer (Eurofins MWG Operon), 0.3 mM dNTPs (Solis BioDyne), 2 mM MgCl_2_, 2 µl Buffer B (Solis BioDyne) and 5 U *Taq* FIREPol (Solis BioDyne). 1 µl primary PCR product was used as template for nested PCR performed in a volume of 20 µl containing 0.25 µM of each primer (Applied Biosystems), 0.2 mM dNTPs (Solis BioDyne), 2 mM MgCl_2_, 2 µl Buffer B (Solis BioDyne) and 1.5 U *Taq* FIREPol (Solis BioDyne). The forward primers for the nested PCR were labelled with fluorescent dyes: 6-FAM for *msp1*F3, NED for MS16. Cycling conditions were as follows: Initial denaturation 95°C for 1 minute, then 30 cycles (primary PCR) or 25 cycles (nested PCR) with 15 seconds denaturation at 95°C, 30 seconds annealing at 59°C and 30 seconds elongation at 72°C plus a final elongation of 5 minutes at 72°C.

All samples negative after the first round of PCR amplification were repeated once. Repeats and all microscopy negative samples (due to an expected lower parasitaemia) were done under similar conditions with the exception that 2 µl DNA solution were used as template for the primary PCR. Capillary electrophoresis was done as described earlier [30]. As the *msp1*F3 nested PCR in general led to more amplification product compared to the MS16 PCR, twice as much MS16 PCR product (2.5 µl of 1∶10 dilution of PCR product) was analysed by capillary electrophoresis.

All alleles were grouped into bins of 3 base pairs according to the possible size of insertions and deletions in coding regions and the repeat size of the microsatellite.

### Data analysis

#### Analysis of 24 h interval bleeds

Sample pairs collected 24 hours apart from the same patient were compared. Sample pairs were excluded from the analysis if antimalarial treatment was given on the first day of paired sampling. Individual genotypes were classified by positivity on each of two consecutive days, leading to two categories for each genotype: one day positive (genotype observed on either day of paired sampling, *n_1_*) and both days positive (*n_2_*). An estimate 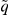 of the detectability *q* was calculated as suggested by Bretscher et al. [31]:
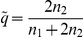
An approximate confidence interval was calculated as follows: CI [*q*±1.96 se(*q*)], where the standard error is:
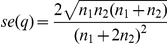
Comparison of detectability between day 1 and day 2 was done by McNemar's exact test for paired data. Detectability was calculated for different age groups of patients, for samples with different MOI (combined over 24 h) and for different parasite densities at day 1. Comparison of different groups was done by nonparametric test for trend across ordered groups. Statistical analysis was performed using STATA® 9.1 statistical analysis software (Stata Corporation, College Station, TX).

## Results

### Parasite detection by light microscopy and PCR-capillary electrophoresis

For the analysis of paired samples collected in 24 h interval, 1019 pairs, equal to 2038 individual blood samples, were eligible. LM diagnosed *P. falciparum* in 398 samples, in 362 samples (91%) this was confirmed by PCR-CE. Additional 210 microscopy-negative samples were *P. falciparum* positive by PCR-CE, leading to a total of 572 samples for which *P. falciparum* genotyping results were obtained. *P. vivax* was diagnosed in 1001 samples by LM, 987 (98.6%) of them were also positive by PCR-CE. Additional 187 samples were positive by PCR-CE, leading to a total of 1174 blood samples with *P. vivax* genotyping results. Thus, sensitivity of LM was lower than that of PCR based diagnosis. Detection of *P. vivax* by MS16 PCR (1161 positive samples) was more sensitive than by *msp1*F3 PCR (1120 positive samples). This difference was significant (McNemar's test: χ^2^ = 9.48, p = 0.002).

### Effect of repeated sampling on observed parasite prevalence

By light microscopy 242 and 563 out of 1019 pairs were positive for *P. falciparum* and *P. vivax*, respectively, at least on either day (Table 1). Observed prevalence did not differ between individual days but it increased when both days were combined. *P. falciparum* parasites were detected in 19.1% of samples at day 1 and 20.0% at day 2 (McNemar's test: χ^2^ = 0.65, p = 0.42). Overall, parasites were detected in 23.7% of sample pairs. Observed prevalence of *P. vivax* was 48.6% on day 1 and 49.7% on day 2 (McNemars test: χ^2^ = 0.88, p = 0.35), and 55.3% when both days were combined.

**Table 1:** Effect of repeated sampling on *P. falciparum* and *P. vivax* prevalence by light microscopy or PCR.

	*P. falciparum*	*P. falciparum*	*P. vivax*	*P. vivax*	*P. vivax*	*P. vivax*
	Microscopy	*msp2* PCR	Microscopy	*msp1*F3 PCR	MS16 PCR	PCR 2 markers^1^
**No. of positive samples**						
1^st^ day pos., 2^nd^ day neg.	39 (16.1%)	21 (6.8%)	57 (10.1%)	39 (6.3%)	49 (7.8%)	40 (6.1%)^2^
1^st^ day neg., 2^nd^ day pos.	47 (19.4%)	28 (9.0%)	68 (12.1%)	56 (9.1%)	58 (9.1%)	55 (8.4%)^3^
Both days positive	156 (64.5%)	262 (84.2%)	438 (77.8%)	521 (84.6%)	527 (83.1%)	557 (85.5%)
**Total no. of pairs positive at least on one day**	**242**	**311**	**563**	**616**	**634**	**652**
**Prevalence (parasite positivity)**						
Prevalence on 1^st^ day (n = 1019 samples)	0.19 (195/1019)	0.29 (283/1019)	0.49 (495/1019)	0.55 (560/1019)	0.57 (576/1019)	0.59 (597/1019)
Prevalence on 2^nd^ day (n = 1019 samples)	0.20 (203/1019)	0.28 (290/1019)	0.50 (506/1019)	0.57 (577/1019)	0.57 (585/1019)	0.60 (612/1019)
Prevalence 2 days combined (n = 1019 pairs)	0.24 (242/1019)	0.31(311/1019)	0.55 (563/1019)	0.60 (616/1019)	0.62 (634/1019)	0.64 (652/1019)
**Detectability** 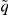 [95% CI]	0.78 [0.74–0.83]	0.91 [0.89–0.94]^4^	0.88 [0.86–0.89]	0.92 [0.90–0.93]^4^	0.91 [0.89–0.93]^4^	0.92 [0.91–0.94]^4^

1 A sample was defined positive for *P. vivax* if any of the two markers *msp1*F3 or MS16 was amplified.

2 These samples are positive on day 1 for at least one marker and negative on day 2 for both markers.

3 These samples are negative on day 1 for both markers and positive on day 2 for at least one marker.

4 Detectability refers to PCR positivity at day 1 versus day 2.

The 1019 sample pairs were genotyped using *msp2* as marker for *P. falciparum* and both *msp1*F3 and MS16 as markers for *P. vivax*. After PCR the number of pairs positive at least on one of both consecutive days increased to 311 for *P. falciparum* and 616 for *P. vivax*. Table 1 summarizes the detection of parasites by PCR on day 1 and 2 of paired samples. Again the observed prevalence of *P. falciparum* as well as *P. vivax* infection did not differ significantly between both days (*P. falciparum*: 27.8% on day 1 vs. 28.5% on day 2, McNemar's test: χ^2^ = 1.0, p = 0.3; *P. vivax*: 58.5% on day 1 vs. 60.5% on day 2, McNemar's test: χ^2^ = 2.21, p = 0.14). When typing results from both days were combined, the observed prevalence increased only marginally: from 28% to 30.6% for *P. falciparum* and from 59.3% to 64.0% for *P. vivax*.

### Effect of repeated sampling on detection of individual clones

**Figure 2:**
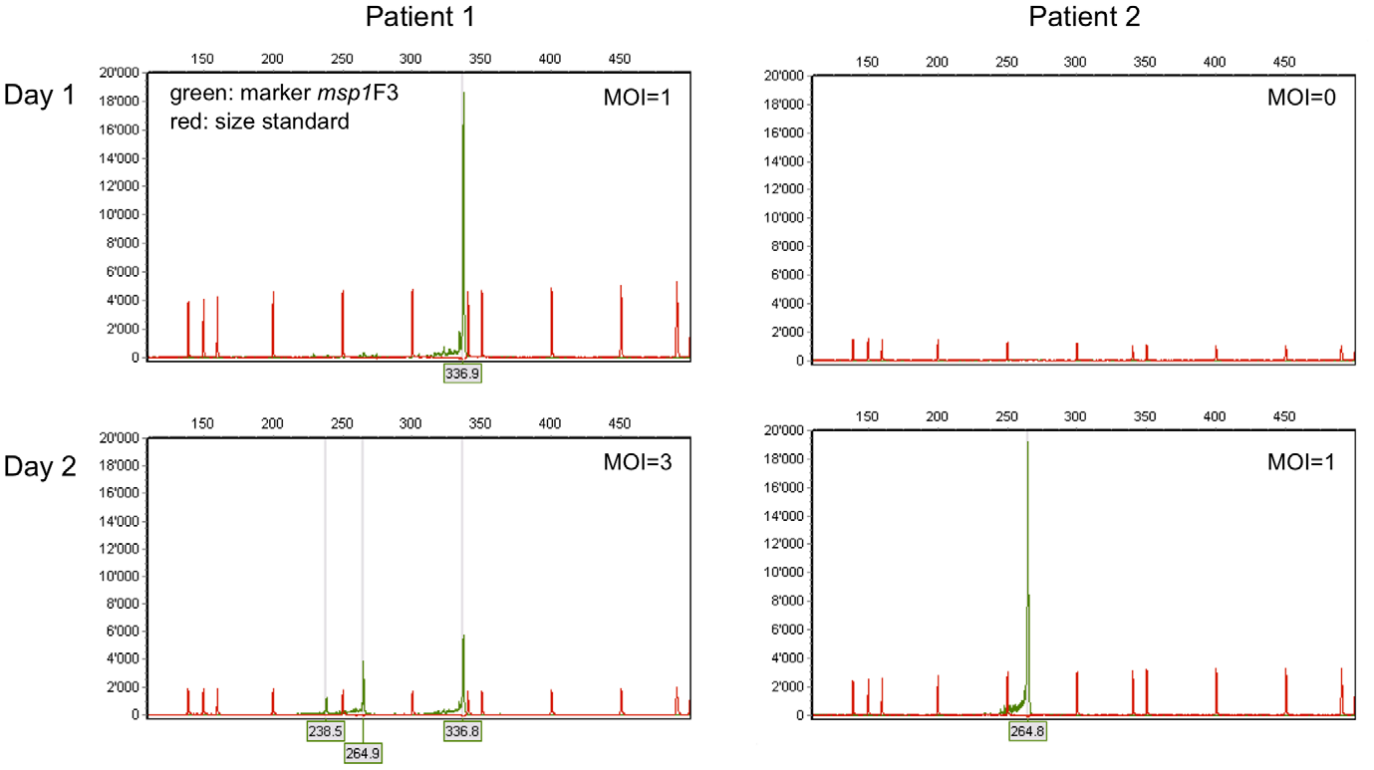
Examples of results obtained on two consecutive days by PCR-capillary electrophoresis. Capillary electrophoresis chromatograms obtained with the *P. vivax* marker *msp1*F3 from two patients on two consecutive days. X-axis: size of PCR product in base pairs. Y-axis: relative fluorescent units. In patient 1 one clone was detected on day 1, and two additional clones were detected on day 2, combined MOI = 3. Patient 2 was negative on day 1, but one clone was found on day 2, combined MOI = 1.

When assessing the persistence of individual alleles on the consecutive days of sampling, considerable turn-over in allele composition was observed (Table 2). Examples from 2 patients are given in Figure 2. For *P. falciparum msp2* 64.7% of the infecting clones were observed on both days. For *P. vivax*, 57.0% of the *msp1*F3 alleles and 43.5% of the MS16 alleles were observed on both consecutive days. These two markers differed slightly in their genetic diversity, with MS16 (virtual heterozygosity *H*_E_ = 0.98) showing greater polymorphism than *msp1*F3 (*H*_E_ = 0.88).

**Table 2:** Effect of repeated sampling on molecular detection of parasite clones and on multiplicity of infection.

	*P. falciparum*	*P. vivax*	*P. vivax*	*P. vivax*
	*msp2*	*msp1*F3	MS16	2 markers combined^1^
**Detection of parasite clones**				
No. clones detected only on day 1	93 (18.0%)	382 (23.8%)	495 (25.2%)	
No. clones detected only on day 2	90 (17.4%)	307 (19.2%)	617 (31.3%)	
No. clones detected on both days	335 (64.7%)	912 (57.0%)	855 (43.5%)	
**Total No. of clones**	**518**	**1601**	**1967**	
**Detectability** 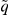 [95% CI]	0.79 [0.76–0.82]^2^	0.73 [0.71–0.75]^2^	0.61 [0.58–0.63]^2^	
**Multiplicity of infection**				
MOI on day 1	1.53	2.31	2.34	2.78
MOI on day 2	1.47	2.11	2.52	2.77
MOI on both days	1.68	2.60	3.10	3.37

1 The highest value for MOI of either marker *msp1*F3 or MS16 was used.

2 Detectability refers to individual genotypes at day 1 versus day 2.

Combining the genotyping results from 24 h bleeds made it possible to assess the effect of repeated sampling on other molecular epidemiological parameters, i.e. prevalence and MOI. In *P. falciparum* combining results from both days lead to a small increase in observed mean MOI to 1.68 compared to an observed mean MOI of 1.52 at day 1. In *P. vivax* the observed mean MOI based on *msp1*F3 increased from 2.21 detected on a single day to 2.60 detected on two days. For the more diverse marker MS16, the observed mean MOI increased from 2.43 to 3.10. When both markers were considered to establish mean MOI, i.e. for each pair the highest number of clones observed was counted, observed MOI increased from 2.78 based on a single day bleed to 3.37 based on results of both consecutive days (Table 2).

Detectability, 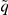, was in the same range for both *Plasmodium* species: for *P. falciparum* clones detectability was 0.79 and for *P. vivax* detectability was 0.73 based on *msp1*F3 marker and 0.61 based on microsatellite MS16.

**Figure 3:**
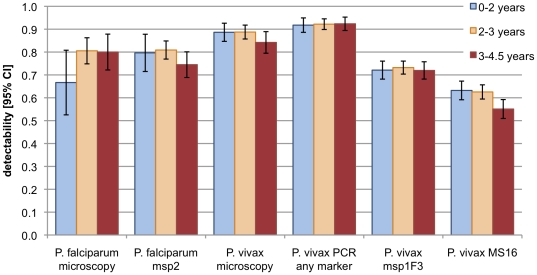
Detectablity of *Plasmodium* infections and parasite clones in different age groups. Values for microscopy and “*P. vivax* PCR any marker” refer to detection of parasites without distinction of clones. Values for the molecular markers *P. falciparum msp2*, *P. vivax msp1*F3 and *P. vivax* MS16 refer to the detection of parasite clones. Larger 95% CI of *P. falciparum* detectability are mainly caused by smaller sample size.

The relationship of clone detectability with age, MOI and parasite density was determined. Figure 3 depicts detectability by age group (0–2 years, 2–3 years, >3 years) for light microscopy and PCR-CE detection. No major difference was observed except for *P. vivax* detection by microsatellite marker MS16, which revealed a slightly lower detectability in children above 3 years compared to younger children (no overlap of 95% CI).

**Figure 4:**
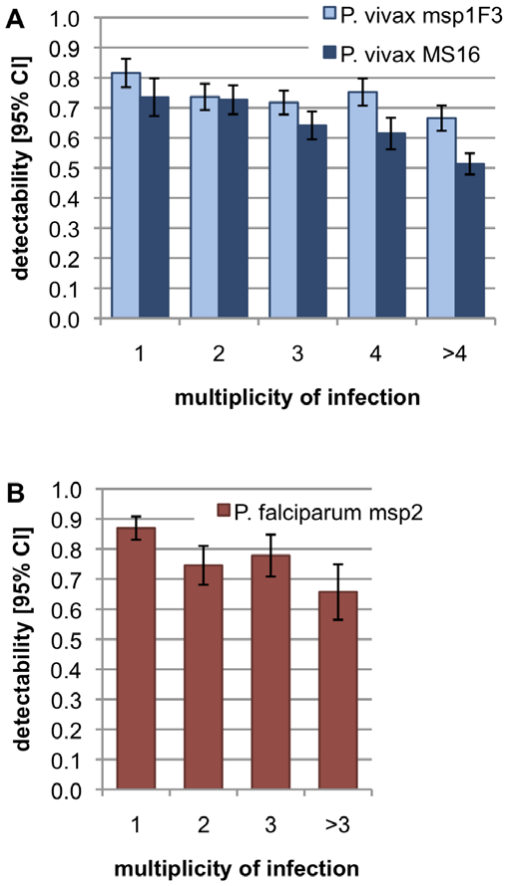
Detectablity of parasite clones vs. multiplicity of infection.

The influence of MOI on detectability was investigated (Figure 4). All markers showed a significant decrease in detectability with increasing MOI (nonparametric test for trend across groups, *P. falciparum msp2*: z = −4.36, *P*<0.001; *P. vivax msp1*F3: z = −3.72, *P*<0.001; *P. vivax* MS16: z = −4.82, *P*<0.001).

**Figure 5:**
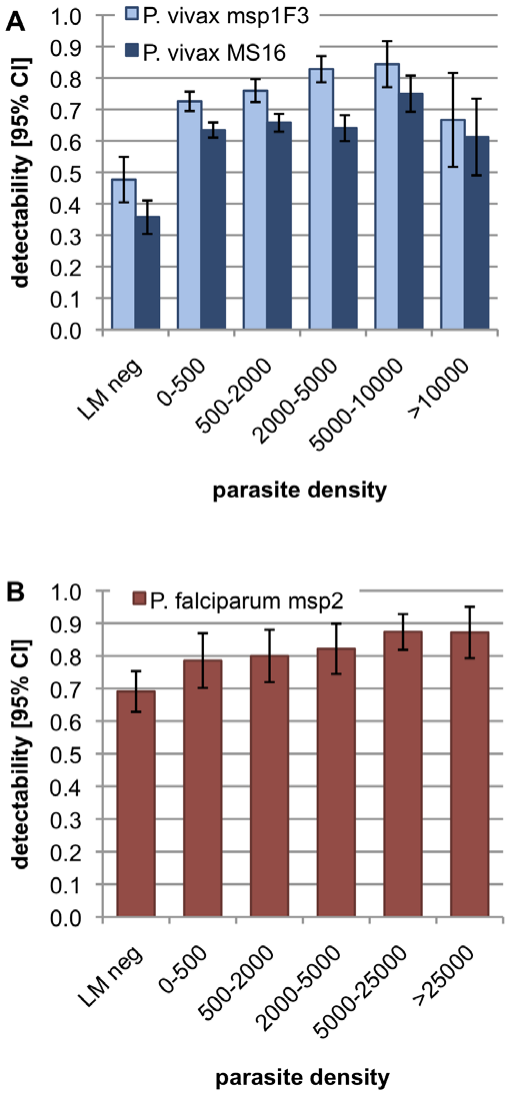
Detectablity of parasite clones in patients harbouring different parasite densities.

Detectability increased with parasite density for both parasite species (Figure 5). *P. falciparum msp2* detectability increased with increasing density. *P. vivax* detectability increased with density until 5000 to 10'000 parasites/µl and decreased thereafter. In samples negative by LM detectability was very low, 0.69 for *P. falciparum msp2*, 0.48 for *P. vivax msp1F3* and 0.36 for *P. vivax* MS16.

## Discussion

Genotyping of malaria parasites has become an integral part of many malariological field studies. Since more than a decade genotyping has been considered imperative for clinical trials of antimalarials performed in endemic countries, The hallmark of PCR correction of clinical trial outcomes is discrimination of new infections versus recrudescences. Quantification of clone detectability at any time point of blood sampling contributes relevant information on the reliability of PCR corrections. Furthermore, the number of newly acquired clones per time interval might be a suitable outcome measurement of antimalarial interventions; the parameter “clone detectability” might also correct this estimate.

Imperfect detectability for *P. falciparum* has been common knowledge, but to date this effect has been quantified only in few molecular epidemiological studies. The detectablity of *P. vivax* clones has been largely ignored due to the reported absence of sequestration. However, the generally lower parasite densities in *P. vivax* compared to *P. falciparum* has potential to contribute to compromised detectablity in a major way. The precise estimation of the detection probabilities of both species, undertaken in the same field study and under perfectly matching experimental conditions, allows assessing the combined effects of parasite sequestration, synchronicity and low parasitaemia and differences among *Plasmodium* species. It is clear that the individual factors, contributing jointly to detectability, cannot be determined by our genotyping approach.

Our analysis of samples collected 24 hours apart revealed limited day-to-day fluctuations in the detection of *P. falciparum* and *P. vivax* infection. This indicates that short-term sampling has only a small impact on prevalence estimates – regardless of whether infections are detected by light microscopy or PCR. For both species the observed prevalence by PCR increased, when 2 days were combined, by less than 10%. A more pronounced difference in prevalence based on one versus two days of sampling was only observed for microscopic detection of *P. falciparum*, where prevalence increased by 24%. It is unclear in how far the effect of parasite synchronization and sequestration add to this discrepancy between PCR and microscopy in *P. falciparum* detectability. These finding suggests that for *Plasmodium* species conducting repeated sampling within 24 h does not substantially increase the observed prevalence.

Detection of individual clones was very high in *P. falciparum* (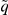 = 0.79). Accordingly, combining genotypes from both days resulted in a small increase in observed mean MOI rising from 1.52 based on one day to 1.68 for both days. Children <5 years have not yet developed a strong immunity to *P. falciparum* [15] and therefore carry high parasite densities (mean parasite density: 2558 parasites/µl), which leads to a better chance to detect by PCR most of the parasite clones present. Recently in a similar study in Ghana blood samples from individuals up to 20 years were repeatedly collected in intervals of 1, 4, 5 and 7 days [31]. Detectability was calculated using the same approach as in our study. Clone detectability was around 0.6 in sample pairs collected 24 hours apart [31].

In contrast to *P. falciparum*, sequestration of late stage parasites has not been reported from *P. vivax*. Despite this biological difference, the detectability of individual parasite clones was lower in *P. vivax* than in *P. falciparum*. A larger number of *P. vivax* clones was only detected on either day for both *P. vivax* markers analyzed. In *P. vivax* parasite densities are generally much lower than in *P. falciparum*, in our study mean *P. vivax* density was 498 parasites/µl compared to the 5-fold higher *P. falciparum* density. Our results suggest that the overall low parasitaemia combined with synchronized replication, as generally seen in *P. vivax*, has a larger impact on detectability than sequestration plus sporadic low parasitaema, as observed in *P. falciparum*.

The number of concurrent *P. falciparum* or *P. vivax* infections had a pronounced effect on detectability: Increasing MOI lead to decreasing detectability. It has been suggested that in multiple-clone infections, clones representing a minority of the total parasite population in a host might escape detection by PCR [32]. In experimental mixtures of DNA from two different *P. falciparum* clones up to ratios of 1∶100, both genotypes were detected by PCR-CE [33], as well as in mixtures of DNA from two different *P. falciparum* clones in a ratio of 1∶5 [34]. However, our observation that detectability decreased with increasing MOI, suggests impaired amplification of minority clones. Hence, the higher mean MOI in *P. vivax* (MOI = 3.37) compared to *P. falciparum* (MOI = 1.68) could be seen as a further reason for the slightly lower detectability in *P. vivax*.

Detectability of the two *P. vivax* markers *msp1*F3 (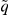 = 0.73) and MS16 (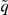 = 0.61) differed. Overall, more samples were positive for *P. vivax* and more clones were detected with the MS16 PCR. This suggests a higher detection threshold of the *msp1*F3 PCR in combination with a lower influence of fluctuations in parasite density on detectability. In addition, a difference in the genetic diversity of these markers could add to the discrepant values: two independent clones are expected to share more often the same *msp1*F3 allele.

Our results highlight that a single bleed does not reflect the full complexity of concurrently infecting *P. vivax* clones. The true MOI of *P. vivax* is underestimated to a greater extent than the MOI of *P. falciparum*. The effect of repeated sampling on prevalence, however, was in the same range in *P. falciparum* and *P. vivax*: for both species 6 to 9% of all infections were missed on any single day. The known biology suggests that low parasite densities should have different causes in *P. falciparum* and *P. vivax*. *P. falciparum* parasites sequester periodically but a clone is absent from the peripheral circulation only if its erythrocytic cycle is tightly synchronized, which according to our data seems not to be the rule. *P. vivax* generally occurs at lower densities, and the timing of a parasite clone within the erythrocytic cycle seems to be well synchronized. These are major differences between both species, but with respect to detectability of parasite clones in the blood stream, both species differ less than previously thought.

In our study participants parasite densities for *P. vivax* dropped with increasing age, while for *P. falciparum* densities remained at the same level [15]. If parasite densities would directly impact detectability, a similar decrease in detectability with increasing age would thus be expected for *P. vivax*. However, we did not observe a clear effect of parasite density or age on detectability. Despite the fact that we did not detect an age trend in detectability in our study participants aged 1 to 4.5 years, this relationship might be different in older individuals. It remains open whether the often observed decrease of prevalence over the entire age range in moderate to high levels of transmission might be due at least partially to lower detectability associated with decreasing parasite densities.

Our *P. falciparum* results were generated by using a similar experimental and analytical approach as in a previous study, conducted in a highly endemic area in Ghana, and results can thus be compared. When all age groups were included in the Ghana study a strong age-dependency of detectability was noted, with a detectability of over 60% in younger individuals and only 10% in adults [24]. Overall, only 35% of all clones present in the host were detected in a single blood sample [18]. In individuals of the same age group as our participants, detectability ranged from 0.51 to 0.55 [31]. This lower detectability in Ghana could be affected by higher malaria endemicity and higher mean MOI. A lower transmission and therefore slower acquisition of immunity in PNG might lead to lower age dependency in detectability. It remains open whether in older children the often-observed decrease of prevalence by age in moderate to high levels of transmission might be due at least partially to lower detectability associated with decreasing parasite densities. Our current results reflect the situation in children harbouring high parasite densities. The situation in adults might be different.

In conclusion, when both 24 h bleeds were combined, we observed an increase in precision of estimates of epidemiological parameters in our study for both *P. falciparum* and *P. vivax*. While the increase in observed prevalence was limited, the effect on detection of individual alleles was more pronounced. Especially in highly endemic countries where most patients carry multiple clone infections repeated sampling substantially increases the precision of observed epidemiological parameters such as MOI. There was surprisingly little difference between the two parasites; just as studies of *P. falciparum* should recognize that they only detect a proportion of infections, the same is true for *P. vivax*.
